# Cardiovascular brain impulses in Alzheimer’s disease

**DOI:** 10.1093/brain/awab144

**Published:** 2021-03-31

**Authors:** Zalán Rajna, Heli Mattila, Niko Huotari, Timo Tuovinen, Johanna Krüger, Sebastian C Holst, Vesa Korhonen, Anne M Remes, Tapio Seppänen, Jürgen Hennig, Maiken Nedergaard, Vesa Kiviniemi

**Affiliations:** 1 Center for Machine Vision and Signal Analysis, University of Oulu, 90570 Oulu, Finland; 2 Oulu Functional Neuroimaging Group, Research Unit of Medical Imaging, Physics and Technology, University of Oulu, 90570 Oulu, Finland; 3 Research Unit of Clinical Neuroscience, Neurology, University of Oulu, 90570 Oulu, Finland; 4 Neurobiology Research Unit, Copenhagen University Hospital, 2100 Copenhagen, Denmark; 5 Department of Diagnostic Radiology, Medical Research Center, Oulu University Hospital, 90220 Oulu, Finland; 6 Department of Radiology, Medical Physics, Medical Center University of Freiburg, Faculty of Medicine, University of Freiburg, 79110 Freiburg, Germany; 7 Center for Translational Neuromedicine, University of Rochester Medical Center, Rochester, NY 14642, USA

**Keywords:** amyloid beta, glymphatic system, Alzheimer’s disease, cardiovascular pulses

## Abstract

Accumulation of amyloid-β is a key neuropathological feature in brain of Alzheimer’s disease patients. Alterations in cerebral haemodynamics, such as arterial impulse propagation driving the (peri)vascular CSF flux, predict future Alzheimer’s disease progression. We now present a non-invasive method to quantify the three-dimensional propagation of cardiovascular impulses in human brain using ultrafast 10 Hz magnetic resonance encephalography. This technique revealed spatio-temporal abnormalities in impulse propagation in Alzheimer’s disease. The arrival latency and propagation speed both differed in patients with Alzheimer’s disease. Our mapping of arterial territories revealed Alzheimer’s disease-specific modifications, including reversed impulse propagation around the hippocampi and in parietal cortical areas. The findings imply that pervasive abnormality in (peri)vascular CSF impulse propagation compromises vascular impulse propagation and subsequently glymphatic brain clearance of amyloid-β in Alzheimer’s disease.

## Teaser

Rajna *et al.* present a non-invasive quantification of cardiovascular impulse propagation within the human brain, and show differences in impulse timing, velocity, and direction in patients with Alzheimer’s disease compared to age-matched healthy volunteers.

**See Bailes and Lewis (doi:10.1093/brain/awab247) for a scientific commentary on this article.**

## Introduction

The most common cause of dementia, Alzheimer’s disease, is imposing an increasing burden on global health care. The canonical neuropathology of Alzheimer’s disease first manifests in the intracerebral accumulation of amyloid-β plaques, which is later accompanied by hyperphosphorylated tau tangles in degenerating neurons.^1^ Unfortunately, clinical trials with inhibitors of the gamma-secretase enzyme or with antibodies against amyloid-β protein aggregates have failed to modify disease progression.^2^ Thus, the growing recognition that amyloid-β accumulation is a necessary but not sufficient condition for Alzheimer’s disease pathology^3^ calls for novel diagnostic and therapeutic approaches targeting the gamut of pathophysiological events in the disease process.

An increasing body of evidence indicates that the progression of dementia is connected to increased blood pressure,^4^ and recent randomized treatment trials support this as aggressive control of blood pressure reduces the incidence of Alzheimer’s disease.^5^^,^^6^ High blood pressure damages blood vessel walls and causes microbleeds and amyloid-β deposition into the perivascular and intramural vessel structures and deteriorates cognitive functions such as memory.^7-9^ Increased pressure stiffens damaged arterial walls and reduces arterial pulsatility, and increases arterial pulse wave propagation speed, which has been associated with increased risk of dementia in vulnerable individuals.^10^^,^^11^ Reduced arterial pulsatility in hypertension reduces the average speed of convection along arterial pathways and can even reverse convection of CSF pulses in perivascular spaces in the glymphatic brain clearance system.^12–14^ A deletion of the AQP4 water channel from the glymphatic system further reduces amyloid-β efflux and increases memory deficits in knock-out mice.^15^

Existing neuroimaging techniques do not yield a non-invasive metric for early and predisposing pathophysiological markers of Alzheimer’s disease. As arterial pulsation properties, such as propagation speed, are predictive of dementia progression, a routine procedure to screen for perturbation of cardiovascular brain impulse speeds would facilitate the detection of an early stage of the Alzheimer’s disease pathway.^11^ The use of ultrafast 10 Hz magnetic resonance encephalography (MREG) presents a method that enables non-invasive quantification of the propagation of human cardiovascular brain impulses that drive blood flow and concomitantly also intracerebral transport and efflux of amyloid-β.^16–18^

In this study, we used the non-invasive MREG technology to investigate the process of cardiovascular brain pulsation in patients with Alzheimer’s disease. We detected striking differences in cardiovascular impulse latency, propagation speed and direction in patients with Alzheimer’s disease compared with age-matched healthy volunteers. The results indicate pervasive alterations in the propagation of the cardiovascular impulse, which together indicate stagnation of impulse propagation within brain of patients with Alzheimer’s disease.

## Materials and methods

### Subjects

Data were compared between 26 (16 female) patients with Alzheimer’s disease aged 57.4 ± 5.7 and disease duration of 3.5 ± 2.2 years, and 31 (18 female) healthy control subjects aged 60.5 ± 4.8 years (Supplementary Table 1). Mini-Mental State Examination (MMSE) score of Alzheimer’s disease group was 22.3 ± 6.3, and 28.6 ± 1.3 for the control subjects. MMSE scores range from 0 to 30, with higher scores denoting better cognitive function.^19^ All the patients were examined by experienced neurologists specialized in memory disorders at the memory outpatient clinic of the Department of Neurology of Oulu University Hospital in Finland. Patients underwent a battery of examinations, including clinical and neurological examinations, laboratory screening tests, neuropsychological examination, and structural and functional MRI of the brain. When deemed necessary for confirmation of Alzheimer’s disease diagnosis, we collected, CSF samples for analysis of the biomarkers amyloid-β_42_, tau and phospho-tau and/or undertook metabolic neuroimaging by fluorodeoxyglucose (FDG) PET. All Alzheimer’s disease group patients met the current NINCDS-ADRDA (National Institute of Neurological and Communicative Disorders and Stroke and the Alzheimer's Disease and Related Disorders Association) diagnosis criteria for probable Alzheimer’s disease.^20^ Age-matched control subjects were interviewed and screened for mild cognitive impairment with the MMSE examination and for depression with Beck's Depression Inventory (BDI). Any history of psychiatric or neurological disorders or recent use of medications affecting the CNS were exclusion criteria for the control group. The study was approved by the Regional Ethics Committee of the Northern Ostrobothnia Hospital District and was conducted in accordance with the Declaration of Helsinki. Participants or their caretakers gave their written informed consent to participate in the study. The recordings in this study exactly match dataset 3 in Tuovinen *et al*.^21^

Because of its exploratory nature, we did not perform a power analysis before performing the study. However, the group sizes were in line with previous experiments using functional MRI in patients with Alzheimer’s disease.^22^^,^^23^ Preprocessing of neuroimaging data included standard automated analytic pipelines, which were agnostic to the diagnostic and demographic characteristics of the data. Data collection and further analyses were not performed blind to the experimental group.

### Measurements

The blood oxygenation level-dependent (BOLD) signal is generally attributed to susceptibility changes in water proton spins inside and around the brain blood vessels due to changes in the local concentration of paramagnetic deoxyhaemoglobin concentration that arise secondary to neuronal activation. Recent *in vivo* microscopy research indicates that the cardiorespiratory brain pulsations also induce pulsatile convection of water, waste and metabolites along the perivascular channels, where BOLD signal also partially originates,^14^^,^^24–27^ within the glymphatic transport. The traversing cardiovascular pulses perturb both peri- and intravascular water spin coherence along the main axis of arterial flow, which is detectable as signal waves with high frequency sampling (repetition time < 300 ms) of the BOLD signal.^16–18^ The critically sampled MREG_BOLD_ signal contains enough information for non-aliasing separation of the three physiological pulsations, i.e. vasomotor, respiratory, and cardiovascular.^17^

All subjects were scanned using a Siemens Skyra 3 T MRI scanner (Siemens Healthineers AG) with a 32-channel head coil. We used an MREG sequence obtained from Freiburg University (J. Hennig). MREG is a single-shot three-dimensional (3D) sequence that utilizes a spherical stack of spirals and undersamples the 3D k-space trajectory.^28^ We used the following sequence parameters: repetition time = 100 ms, echo time = 36 ms, field of view = 192 mm,^3^ voxel size = 3 mm^3^ and flip angle = 5°. MREG data were reconstructed by L2-Tikhonov regularization with λ = 0.1, with the latter regularization parameter determined by the L-curve method.^29^ The resulting effective spatial resolution was 4.5 mm full-width at half-maximum (FWHM). MREG image reconstruction also included a dynamic off-resonance in k-space (DORK) method, which corrects prior to preprocessing for scanner warming and for respiration-induced dynamic B_0_-field changes.^30^ An example MREG time frame after reconstruction and masking is presented in Supplementary Fig. 1.

Anatomical high-resolution T_1_-weighted MPRAGE (repetition time = 1900 ms, echo time = 2.49 ms, inversion time = 900 ms, flip angle = 9°, field of view = 240 and slice thickness = 0.9 mm) images were obtained for co-registration of the MREG data to each subject's own anatomy, followed by resampling into the T_1_ Montreal Neurological Institute (MNI 152) 4 mm^3^ standard space. During the 5-min MREG resting state study of 2961 volume frames, subjects were instructed to lie still in the scanner with their eyes open, while fixating their gaze on a cross, presented on a video monitor. Soft pads and ear plugs were fitted over the study subjects’ ears to dampen auditory stimuli and minimize head motion.

### Preprocessing

MREG data were preprocessed with an FSL pipeline.^31^ The data were high-pass filtered with cut-off frequency of 0.008 Hz (125 s) and 80 time points (8 s) were removed from the beginning of the time series to minimize T_1_-relaxation effects. Motion correction was performed using FSL MCFLIRT.^31^ There was no significant difference in absolute or relative head motion parameters between subject groups (Supplementary Table 1), and to the extent measurable by FSL MCFLIRT,^31^ they did not significantly contribute to differences in cardiovascular data between our subject groups (see dataset 3 in Tuovinen *et al*.^21^). Brain extraction was carried out with FSL BET^31^ using the following parameters: fractional intensity = 0.1–0.25, threshold gradient = 0.1–0.22, with neck and bias-field correction option. Spatial smoothing was carried out using 5 mm FWHM Gaussian kernel. No additional post-processing for B_0_-field corrections were made.

Time series data were filtered to exclude frequencies lower than the cardiovascular signals using AFNI^32^ 3dTproject with a passband of 0.6–5.0 Hz. Brain data were visualized with FSLeyes,^33^ matplotlib^34^ and MATLAB (The MathWorks Inc, MA, USA).

### Cardiovascular pulse arrival

**Figure 1:**
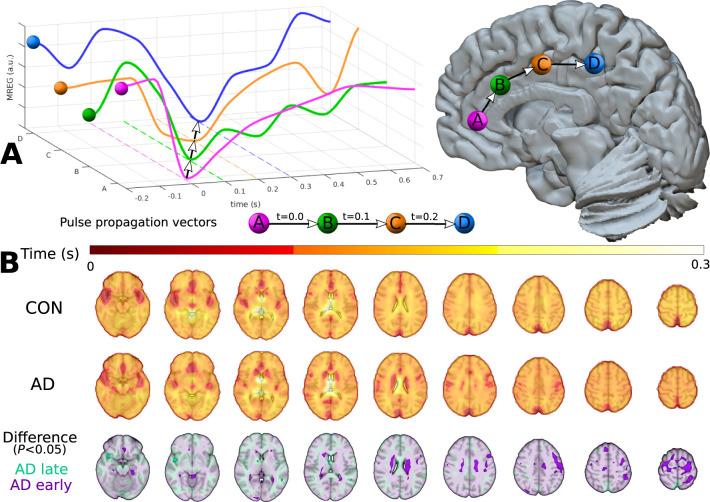
**The cardiovascular pulse propagation vector and voxel-wise pulse arrival times relative to the ACA.** (**A**) Schematic overview of cardiovascular pulse propagation in human brain as functions of time and spatial location. The cardiovascular impulse induces a sharp drop in the MREG signal that moves through the brain as a wave. Optical flow algorithm follows this drop to calculate the local propagation speed (see ‘Materials and methods’ section’. (**B**) Mean cardiac pulse arrival times with ACA reference [MNI (0, 30, 3)] in seconds for Alzheimer’s disease (AD) and control groups. The bottom row shows their difference (*P < *0.05, FDR-corrected), where lower pulse arrival latency is shown in purple (AD early) and greater latency is shown in turquoise (AD late).

**Figure 2:**
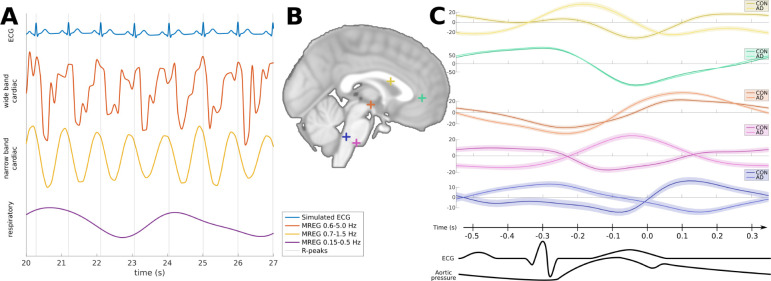
**Examples of the MREG signal shape.** (**A**) Physiological MREG signals at the ACA in QRS synchronization with simulated ECG plots. From *top* to *bottom*: simulated ECG signal matching measured QRS timing, wide band cardiac MREG signal used in this study (0.6–5.0 Hz), narrow band MREG cardiac signal (0.7–1.5 Hz), and respiratory MREG signal (0.15–0.5 Hz). Vertical axes are in arbitrary units, and only MREG signals are comparable. (**B**) Cardiovascular MREG signal examples of the 0.9 s cardiac cycle used in this work at several representative locations in the brain midline. Average signals are separately shown for control and Alzheimer’s disease group with 95% CI in the background. Vertical axes are MREG signals in arbitrary units.

The time of the cardiovascular pulse arrival in brain was measured from the ECG-verified QRS-complex event timing and the pulse dip in the MREG signal measured at the beginning of A3 segments of anterior cerebral arteries (ACA) defined at two neighbouring voxels centred at midline MNI coordinate (0, 30, −3). An example for the signal shape is shown in Fig. 1A, with additional details in Fig. 2A. Consistently in each cardiac cycle, we observed an MREG signal dip in the ACA following the QRS. The consistency of arrival was visually confirmed with narrow band cardiac MREG data (0.7–1.5 Hz), and the more accurate timings were measured with the wide band cardiac data (0.6–5.0 Hz) used in all further analyses (Fig. 2A).

**Figure 3:**
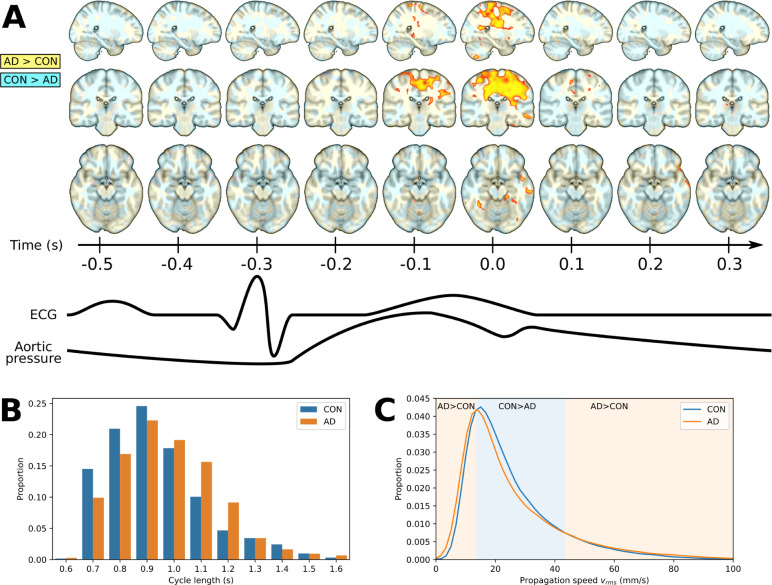
**Speed (*v*_rms_) differences in Alzheimer’s disease and control groups, the distribution of cardiac cycle length, and the whole brain average of propagation speed.** (**A**) The 3D time lapse video of the differences (background colour) and significant differences (*P < *0.05, FDR-corrected) between Alzheimer’s disease and control groups in speed magnitude (*v*_rms_) of the cardiovascular impulse propagation in a dynamic 3D plot (see Supplementary Video 1 and Supplementary Fig. 4 for full brain coverage). (**B**) Median 0.9 s cycles were chosen for optical flow analysis based on the similar heart rate distributions from Alzheimer’s disease subjects and controls. (**C**) Distribution of mean cardiovascular impulse propagation speed (*v*_rms_) across the whole brain data. Background colours separate the three zones; low (0–14 mm/s, *P < *3.0 × 10^−4^) and high speeds (44–100 mm/s, *P < *1.7 × 10^−19^) predominate in Alzheimer’s disease, while mid-range speeds (15–43 mm/s, *P < *5.1 × 10^−8^) speeds predominate in controls.

We used the signal minimum at the ACA as a trigger to segment individual cardiac cycles in the MREG data. This segmentation was used for all further analyses. As explained in the ‘Latency of cardiovascular pulse arrival in Alzheimer’s disease’ section, we selected beats of 0.9 s duration from 17 393 cardiac cycles (*n_CON_*0.9=* *1985; *n_AD_*0.9=* *2022; Fig. 3B). For visualization (Fig. 2B), we selected five physiological points of interest in the brain midline and present the mean wide band cardiac signal at those points for the contrast of control subjects and the Alzheimer’s disease group. Figure 2C also shows the 95% confidence intervals (CI), and reveals that different locations have distinct patterns of signal variability (e.g. signal shape and variance). Since our findings were focused around the well-defined pulse arrival time (*t *=* *0.0 s), we also show in Supplementary Fig. 3 that directional changes of pulse propagation in the Alzheimer’s disease group are not dependent on the selected cardiac cycle length.

As shown in Fig. 1B, we defined the voxel-wise pulse arrival latency as the time difference between the signal dip (local minima) at the ACA from that in every other voxel in each cardiac period (Fig. 2C). The search for the local minima was done separately for each cardiac period. First, the cardiac period was extended with a copy of itself, upsampled by a factor of 10 with third-order spline interpolation. Values over the manually selected time threshold of 0.6 s (technically falling after the next QRS signal) were designated as preceding the ACA arrival, i.e. a negative time difference. Each voxel was assigned a timing value (in s) for every 0.9 s cardiac cycle in this manner, and differences were found between controls and Alzheimer’s disease subjects using our extended method based on FSL randomize.^35^ Since this is circular data, depending on the time threshold by which minima are classified into current or next cardiac cycles (e.g. −0.3 s = 0.6 s in this regard), estimates of latencies lying close to the threshold value will be less accurate due to blurring. Therefore, we focused on data after ACA pulse arrival.

### Pulse propagation speed

3D multi-resolution optical flow of cardiovascular pulse propagation wavefronts were calculated for each subject^18^ (Supplementary Fig. 2). For the wavefront extraction, a minimal peak distance threshold of 0.6 s was used, the multi-resolution depth parameter was set as 3, and the maximal voxel distance considered in the flow estimation was 2 at each resolution level. The result of this analysis is a 3D vector field for every time point, being non-zero only at the instant when the cardiovascular pulse wavefront passes through the given voxel. The vectors are an estimate of cardiovascular pulse wavefront propagation speed (velocity). The Euclidean norm of this velocity was used in comparing propagation speeds between groups (Equation 1) (Fig. 3A and Supplementary Fig. 4):

$v_{\text{rms}}\;=\sqrt{v_{x}^{2}+v_{y}^{2}+v_{z}^{2}}$(1)

### Direction of pulse propagation

The direction (3D unit vector **u**) of maximal difference in the cardiovascular pulse propagation speeds was found for each phase of the cardiac cycle according to the following equation. Here, for every voxel location *xyz* in space, the group difference between the projections to a 3D line defined by unit column vector ***u***(*ρ*,*θ*) was maximized: (2)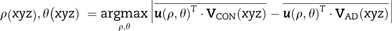 where *ρ* is the polar angle, *θ* is the azimuthal angle of spherical coordinates of **u**. **V**_CON_(*xyz*) is the 3×*n* flow matrix for controls, and **V**_AD_(*xyz*) refers to Alzheimer’s disease subjects, with each column being a propagation vector at location *xyz*, and *n* being the number of cardiac cycles recorded. The multiplication dot symbol designates matrix multiplication, the overline indicates the mean of the vector and vertical bars the absolute value.

**Figure 4:**
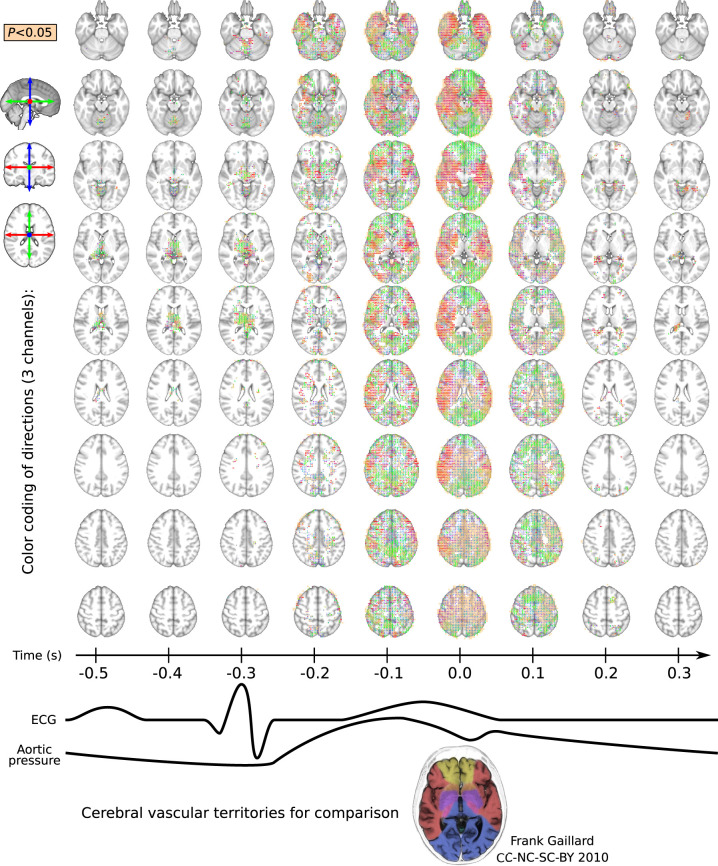
**Time-lapse image over the entire brain at 0.1 s time resolution indicating the direction u of maximal difference in cardiovascular impulse propagation speed (*v_u_*) between control and Alzheimer’s disease groups.** The directions are marked by unit sticks and colour, and *P < *0.05 differences are indicated with beige voxel background. The maximum area coverage of impulse abnormality in Alzheimer’s disease coincides with the cardiovascular impulse arrival to the brain at 0.6 s, i.e. some 0.3 s after the ECG R-peak. The maximal *v_u_* change follows the general flow directions in the main arterial territories the brain: note the hand drawn cerebral vascular territory map on the bottom for comparison. During brain impulse diastole the most Alzheimer’s disease differences are localized in central thalamic structures.

The optimization of Equation 2 was implemented with the Nelder-Mead algorithm, initialized by a coarse search on an equidistant 10 × 10 grid. Here, calculations were performed only when flow vectors were available for at least 10% of the cardiac cycles. The direction of maximal difference was found for each cardiac phase independently (Fig. 4). We note that the optimization problem is symmetric, since every unit vector ***u***(*ρ*,*θ*) has the same value for Equation 2 as *−****u***(*ρ*,*θ*). This makes the result for the Alzheimer’s disease group being bigger or smaller than control values mathematically indifferent, depending only on the choice between ***u*** and *−****u*** vectors. However, the propagation speed difference is maximal along the projection of the same 3D line defined by both ***u*** and *−****u***, which was used in the ‘Results’ section.

**Figure 5:**
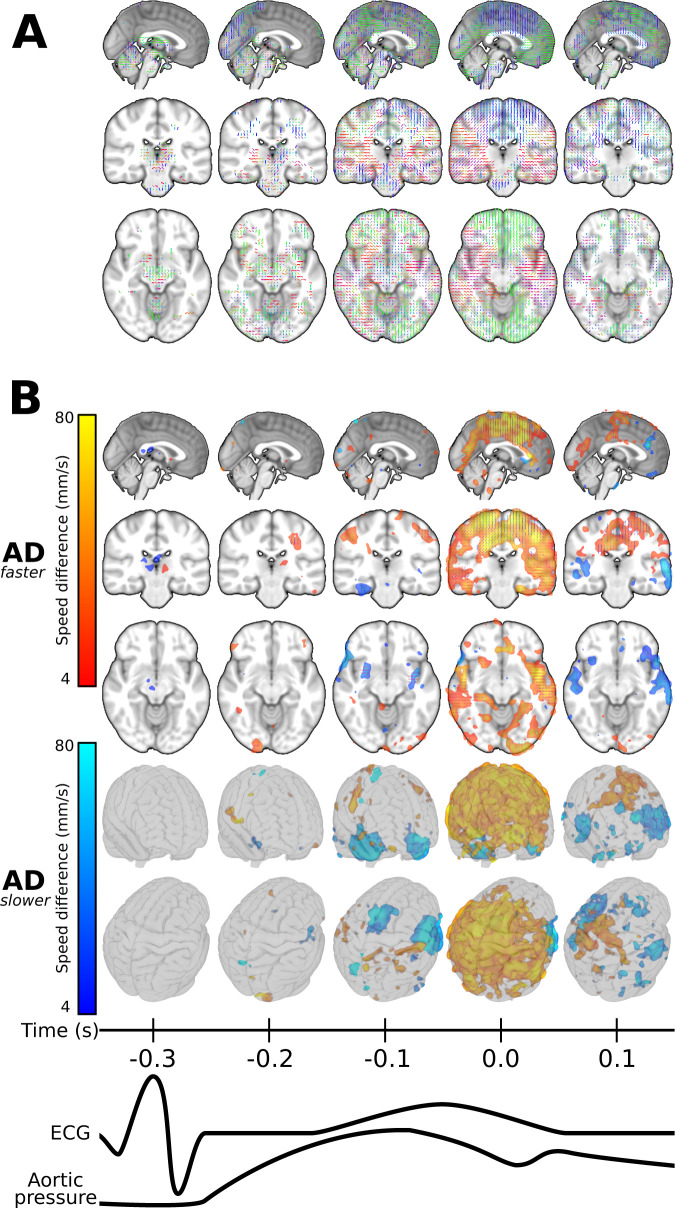
**Faster and slower pulse propagation speed (*v_u_*) in Alzheimer’s disease.** Selected 3D planes [MNI: (0, −25, −11)] showing (**A**) the directions of maximal *v_u_* difference as in Fig. 4, and (**B**) types of difference and their coverage (*P < *0.05, FDR-corrected) in those directions. Zones: Alzheimer’s disease (AD) propagation faster (red–yellow), Alzheimer’s disease propagation slower (blue–light blue). The colour intensities in **B** represent the amount of difference in propagation speed along directions in **A** on the same scale as in Fig. 6A (4–80 mm/s). The two transparent glass brain projections indicate the wide area coverage of *v_u_* changes detected. The complete cardiac cycle is presented in Supplementary Video 2 and Supplementary Fig. 5.

**Figure 6:**
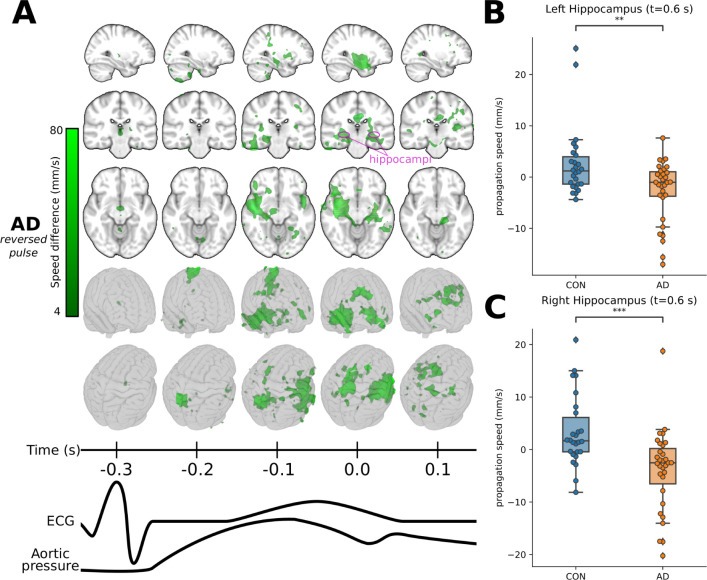
**Reversed pulse propagation direction in Alzheimer’s disease.** (**A**) Zone of opposite propagation direction in Alzheimer’s disease brain where the difference is significant (in green, *P < *0.05, FDR-corrected). In this volume, maximal *v_u_* differences have opposite signs (direction). The volume is shown in selected 3D planes [intersecting at MNI: (30, −25, −11)] and in a glass brain to demonstrate its spatial extent. The colour intensities represent the magnitude of the group difference in propagation speed along the same directions as in Figs 4 and 5A and of the same scale as in Fig. 5B (4–80 mm/s). The complete cardiac cycle and **u** directions are presented in Supplementary Video 2 and Supplementary Fig. 6. (**B** and **C**) Multi-level statistical analysis for hippocampal regions. Distribution of subject-wise mean propagation speed (*v_u_*) in the left and right hippocampi differ significantly for (**B**) the left hippocampus (*P = *3.34 × 10^−^^3^) and (**C**) the right hippocampus (*P = *3.95 × 10^−4^) to Welch’s *t*-test. Hippocampi are marked with pink circles on the axial images in **A.**

Upon obtaining **u**, we calculate for each voxel the *v_u_* speed scalars, which are projections of the propagation velocity **v** to **u** direction (Supplementary Fig. 2B). Where control group and Alzheimer’s disease *v_u_* means had the same sign, their absolute values defined the larger of the two, and both were interpreted as positive speed (Fig. 5 and Supplementary Fig. 5). No such interpretation is possible where the sign of the means differed. The corresponding areas were intuitively marked with opposite/reversed propagation direction in Fig. 6 and Supplementary Fig. 6.

Spatial correlation between *v_u_* differences and the group level grey matter atrophy map (see dataset 3 in Tuovinen *et al*.^21^) were calculated with fslcc^31^ and are presented in Supplementary Table 2.

### Statistical considerations

We analysed and compared cardiovascular cycles of the control and Alzheimer’s disease subject groups. To optimize comparability, we selected a length of 0.9 s for cardiac cycles, given the accuracy permitted by the MREG 10 Hz sampling rate. On one hand, this serves the purpose of making directly comparable individual phases of the cardiac cycles without interpolation. On the other hand, this decision also increases the mean distance of the cardiac cycles from each other, reducing temporal autocorrelation originating from phenomena other than similarity of cardiac cycles themselves. Temporal autocorrelation of the MREG cardiac signal drops promptly (0.3 s lag) to near zero, and repeatedly rises with multiples of the cardiac cycle lengths to a weak correlation of ∼0.2. We also investigated the relationship of inter- and intrasubject correlation with the variation partitioning coefficient (*ϱ*), resulting in ‘poor’ intrasubject similarity (*ϱ *< 0.4) for most voxels according to an established evaluation scale.^36^

We pooled both subject groups together and calculated

$\varrho =\frac{\sigma_{g}^{2}}{\sigma_{g}^{2}+\sigma_{s}^{2}}$ (3)

for every voxel where data were available. Here, $\sigma_{g}^{2}$ is the intersubject variance between subject means and $\sigma_{s}^{2}$ is the pooled intrasubject variance of cardiac cycle samples to subject means. This analysis showed that considering all cardiac phases along with *v*_rms_ and *v_u_* statistics, only 2.2 ± 2.0% of brain voxels had *ϱ *≥  0.4, and 0.1 ± 0.1% of brain voxels had *ϱ *≥* * 0.5. The sparse voxels with high *ϱ* had a random spatial distribution, such that we cannot pinpoint any particular region that is highly disposed to intrasubject similarity.

With the exceptions of whole brain propagation speed distribution (Fig. 3C) and the region of interest multi-level analysis (Fig. 6B and C) we used a customized non-parametric permutation test to compare group means, which is a slightly modified version of the FSL randomise algorithm.^35^ To reflect our hierarchical data structure, the permutation (random relabelling) was only performed on the highest level (i.e. subjects), while the assignment of lower level properties (individual cardiac cycles) was constant. This is in line with best practices in non-parametric bootstrapping of hierarchical data.^37^ To reject the null hypothesis, the unshuffled t-statistic needed to exceed 95% of random permutations voxel-wise, and to control the false discovery rate (FDR), each voxel-wise statistic also needed to exceed the spatially global threshold (95 percentile of the *t*-statistic distribution of the whole brain) for the given random permutation (see family-wise error rate control in FSL randomise).

In Fig. 6B and C, we show results for multi-level region of interest analysis in bilateral hippocampus^38^ with top-level statistic inference on subject-wise means. The presented statistics give a firm foundation for our main findings.

### Data availability

Difference in propagation speed (*v_u_* in mm/s) of areas with faster, slower, and reversed pulse propagation in Alzheimer’s disease, as in Figs 5 and 6A, are provided in the Supplementary material in NIfTI format: AD_faster.nii.gz, AD_slower.nii.gz, and AD_reversed.nii.gz, respectively. Directions (**u**) of maximal difference in Alzheimer’s disease at cardiac impulse arrival as in Supplementary Fig. 3B are also provided in the Supplementary material in NIFTI format: AD_directions.nii.gz. Further data supporting the findings of this study and custom routines used for analysis are available from the corresponding author (Z.R.) upon request.

## Results

### Latency of cardiovascular pulse arrival in Alzheimer’s disease

Dynamic pressure gradients guide and promote the movement of CSF in brain, such that stagnation of interstitial fluid (see ‘Introduction’ section) may promote amyloid-β accumulation. Therefore, we set about to monitor the propagation of brain-wide cardiovascular impulse wavefronts detectable in MREG data that propagate from arteries into grey, white matter and extend simultaneously into CSF via connections with perivascular spaces. The impulses initiate upon arrival of the cardiovascular impulse through the cerebral arteries. The cardiovascular impulses induce a wavefront of MRI signal drop inside arterial vessels and in surrounding perivascular spaces formed by a momentary water proton spin incoherence wriggle from convective pressure thrust^14^^,^^16^^,^^39^ intertwined with accompanying BOLD contrast and spin density effects. Fig. 1A and Supplementary Fig. 2 show schematic examples of the pulse propagation vector (see ‘Materials and methods’ section).

The mean cardiovascular pulse arrival time in brain followed 290 ms (276–305 ms, 95% CI) after the ECG R-peak in healthy control subjects. We measured this arrival time from the delay between the ECG-verified QRS-complex event and the subsequent pulse dip in the MREG signal at the beginning of A3 segments of ACA defined at two neighbouring voxels centred at a midline coordinate (0, 30, −3) of the MNI standard brain. For simplicity of presentation, we used a standard 0.3 s latency from R-peak, with the ACA pulse arrival set at *t *=* *0.0 s in the subsequent MREG data analysis.

From 17 393 cardiac cycles recorded in the MREG data with 10 Hz temporal resolution, the mode and median of physiologically verified cardiac pulse length were 0.9 s in both study groups (*n_CON_*0.9=* *1985, *n_AD_*0.9=* *2022). Subjects experienced a mean (SD) of 70 (40) pulses of 0.9-s length per recording, which corresponded to a mean pulse spacing of 4.23 ± 7.5 s over the 5 min scans (see ‘Statistical considerations’ section). The distributions of measured pulse lengths proportions (Fig. 3B) were roughly similar in the two subject groups, given the 10 Hz temporal resolution of the functional MRI sequence.

Compared to the arrival time at the ACA, the group mean maps (Fig. 1B) show the briefest latency to the cardiovascular pulse arrival in the major cerebral arteries and the venous sagittal sinus. This pulse arrives in grey matter 100–200 ms later and in white matter after a further delay of about 100 ms. The maximal mean latency of 0.3 s occurred at the central CSF areas and corpus callosum (Fig. 1B). In the Alzheimer’s disease group, the cardiovascular impulse latency was briefer in basomedial temporal areas including the hippocampi, periventricular white matter, proximal ACA, and superior frontal cortex on the left side (*P < *0.05, FDR corrected) (Fig. 1B). The Alzheimer’s disease group showed relatively longer impulse arrival latency in areas proximal to the bilateral medial cerebral artery (MCA), distal ACA, and the sagittal sinus (Fig. 1B; *P < *0.05, FDR corrected).

### Dynamic alteration of pulse propagation in Alzheimer’s disease brain

An altered anatomical environment for fluid flow might be either the cause or consequence of disease-related changes in the propagation speed of pressure gradient pulses in brain. Therefore, we made a group comparison of the speed of cardiovascular pulse propagation (*v*_rms_), which is the magnitude of the 3D propagation velocity vector, i.e. independent of direction. As shown in Fig. 3C, the average *v*_rms_ distribution in living brain, the most likely *v*_rms_ for a given time point, is ∼15 mm/s in the Alzheimer’s disease and control groups. However, the speed distribution had high variance in the population of Alzheimer’s disease patients, whereas the range in the control group had a more restricted distribution of values. Intermediate mean speeds (15–43 mm/s) were more likely to occur in the control group (*P = *5.07 × 10^−^^8^), whereas members of the Alzheimer’s disease group were more likely to show low speeds (<15 mm/s; *P = *2.97 × 10^−^^4^) or high speeds (>43 mm/s; *P = *1.63 × 10^−^^19^) compared by two-sided paired *t*-tests.

The 0.9 s median (and mode) heart cycles were further compared phase by phase to pinpoint the temporal and spatial locations of *v*_rms_ differences (Fig. 3A). The impulse propagation speed (*v*_rms_) in the Alzheimer’s disease group differed significantly as a function of the position in the cardiovascular cycle from corresponding results in the controls. The relative increases in *v*_rms_ (*P < *0.05, FDR corrected) occurred around the time of cardiac impulse arrival in brain, i.e. 0.2–0.3 s after the ECG R-peak (Fig. 3A, at *t* = −0.1 s and *t *=* *0.0 s).

The increased propagation speed in the Alzheimer’s disease group matches the shorter latency of the peak arrival time, as seen in Fig. 1B. The brain regions showing lower *v*_rms_ were mainly adjacent to major arteries and the sagittal sinus (difference not significant). There were also foci in the mesial temporal lobe in the Alzheimer’s disease group with relatively low *v*_rms_ across the entire cardiac cycle. As an indication of the dynamic nature the pulse propagation, the Alzheimer’s disease group showed zones of increased or decreased *v*_rms_ in periventricular areas across the cardiac cycle. For example, we saw initial *v*_rms_ increases in the periventricular white matter trigone at *t* = −0.1 s and *t *=* *0.0 s, followed by decreases in *v*_rms_ (Supplementary Fig. 4).

### Directions of maximal impulse speed difference in Alzheimer’s disease

To investigate more closely the anatomical underpinnings of the altered pulse propagation in Alzheimer’s disease, we also analysed the directions of the velocity vectors, and their changes in Alzheimer’s disease. This analysis indicated overall matching with arterial territories and revealed some regions with reversed propagation direction in Alzheimer’s disease. We made voxel-wise calculations of the direction of the largest difference between projections of cardiac pulse propagation vectors in the Alzheimer’s disease group compared to controls. We denote this direction with its unit vector **u** and the velocity vector of pulse propagation with **v**, where the projection of **v** on **u** indicates the speed, *v_u_* (see ‘Materials and methods’ section). The directional maps of **u** in 0.1 s steps over the entire cardiac cycle are shown in Fig. 4 (*v_u_* different *P < *0.05, FDR corrected). As with *v*_rms_, the largest *v_u_* differences in the Alzheimer’s disease group were also coincident with the cardiac impulse arrival, i.e. 0.3 s after the ECG R-wave.

At the time of cardiac impulse arrival, the **u** directions of maximum difference respected cerebral irrigation territories along the mean flow direction of the major brain arteries, as indicated by the colour of unit sticks in Fig. 4. There were in the sagittal orientation of the anterior and posterior cerebral arterial territories (green sticks), the middle cerebral artery territory (red sticks), and in the upper pericallosal artery territory in the rostro-caudal orientation (blue sticks) (Figs 4 and 5A; *P < *0.05). In arterial watershed areas, the directional changes bend smoothly along the transition zones (Fig. 4, purple, orange, and cyan colours, *P < *0.05). In the thalamic region and in the cerebral ventricles, the maximal *v_u_* group difference occurred during brain impulse diastole, some 0.1–0.4 s after the cardiovascular impulse arrival.

At the very moment of cardiovascular impulse arrival in brain, 0.3 s after the ECG R-peak (*t *=* *0.0 s), *v_u_*, the speed of impulse propagation in the Alzheimer’s disease group exceeded that in controls over a broad domain of upper/posterior brain areas (Fig. 5B, red–yellow zone, *P < *0.05). The *v_u_* was lower in the Alzheimer’s disease group in mesiotemporal and basal areas, thus in the territories of the middle cerebral and cerebral arteries (Fig. 5B, blue–light blue zone, *P < *0.05).

### Reversed impulse waves in Alzheimer’s disease brain

In addition to being abnormally fast or slow, abnormal pulse propagation in the Alzheimer’s disease group could also manifest in a completely reversed direction of propagation, which may be of particular relevance to understanding Alzheimer’s disease pathology. Peaking between *t* = −0.1 s and *t *=* *0.1 s, we see zones of reversed impulse propagation (*v_u_*) mainly on the right side in periventricular areas, mesiotemporal structures such as hippocampus, anterior ventromedial structures, and in posterior and lateral thalamic regions of the Alzheimer’s disease group (Fig. 6A, green zone, *P < *0.05). In Fig. 6B and C, we present the region of interest multi-level analysis showing reversed direction of *v_u_* in bilateral hippocampus in the Alzheimer’s disease group compared with controls.

**Figure 7:**
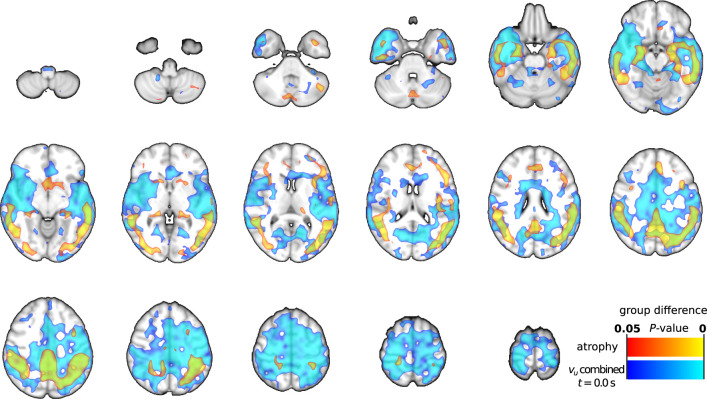
**Spatial overlap of grey matter atrophy and most correlating results.** Grey matter atrophy group differences (*P < *0.05) are presented in red–yellow, and combined optical flow (*v_u_*) result at *t *=* *0.0 s (group differences, *P < *0.05) in blue–light blue colours. Combined *v_u_* results are the spatial combination of areas with significantly increased, decreased, or reversed cardiovascular impulse propagation in Alzheimer’s disease (Figs 5 and 6A). Spatial correlation is at the maximum of 0.16 at presented *t *=* *0.0 s (Supplementary Table 2).

Spatial correlations between the group level grey matter atrophy map (see dataset 3 in Tuovinen *et al*.^21^) and *v_u_* difference maps (Figs 5 and 6A) were poor: maximum of 0.16 with all *v_u_* results combined (Supplementary Table 2). Spatial overlaps of grey matter atrophy and combined (increased, decreased, and reversed) *v_u_* at maximum spatial correlation (*t *=* *0.0 s) are presented in Fig. 7. Notable partial overlaps were found in the temporal and parietal lobes. This suggests that atrophy can play a role in the observed changes of impulse propagation in Alzheimer’s disease in the areas overlapping with atrophy.

For each type of the three abnormal pulse propagation patterns identified (Figs 5 and 6A) in the contrast of Alzheimer’s disease with control subjects, we summarized the proportions of affected brain volumes at each phase of the cardiac cycle. Table 1 presents the brain volume proportions where the propagation speed (*v_u_*) difference between Alzheimer’s disease and control subjects was at least 4 mm/s in the difference-maximized direction **u** (*P < *0.05). We also compared the distribution of brain voxels with abnormal pulse propagation patterns with typical PET findings of increased amyloid-β accumulation in early Alzheimer’s disease patients (Aβ_ROI_).^40^ We found similar associations in regional amyloid-β findings as in whole brain, with two notable exceptions: reversed pulses dominated in the Aβ_ROI_ just before and at pulse arrival (22.4%, 12.4%), while the volume proportion for delayed pulses was larger after impulse arrival into the brain (11.1%). The maximum overlap with the amyloid-β volume (47.6%) was with the increased pulse propagation speeds occurring coincident with the pulse arrival. PET is more sensitive to detect parenchymal amyloid-β depositions compared to intravascular amyloid-β, therefore overlapping findings may rather reflect paravascular than intravascular amyloid-β involvement in the process.

**Table 1:** Area ratio of zone types with significant difference in Alzheimer’s disease

Zone type	−0.3 s	−0.2 s	−0.1 s	0.0 s	0.1 s
**Area ratio of zones compared to the whole brain**					
AD faster	0.333%	3.78%	7.35%	42.0%	11.0%
AD slower	0.522%	1.22%	6.38%	2.28%	9.64%
AD reversed	1.93%	5.30%	11.4%	7.38%	8.24%
**Area ratio of zones intersecting with Aβ_ROI_**					
AD faster	0.127%	3.29%	5.06%	47.6%	9.82%
AD slower	0.063%	1.58%	4.69%	2.72%	11.1%
AD reversed	0.697%	6.90%	22.4%	12.4%	4.88%

Time points are relative to the cardiovascular pulse arrival into the brain. For time points −0.5 s, −0.4 s, 0.2 s, and 0.3 s the area ratio is under 1% for all zones. Aβ_ROI_ = PET findings of increased amyloid-β accumulation in early Alzheimer's disease.

## Discussion

Our optical flow analysis^18^ of ultrafast MREG brain cardiovascular impulse data indicates that the cardiovascular brain impulse is significantly abnormal in Alzheimer’s disease. Overall, the distribution of brain cardiovascular impulse movement (note, not physical flow) was significantly shifted towards low and high-speed extremes compared to the distribution in healthy controls. The maximal change in impulse spread followed the direction of territorial blood flow along major cerebral arteries. Both magnitude and direction of the impulse speed showed differential changes along the vascular trees, with speed reductions in proximal areas, versus increases in peripheral territories with smaller arteries. Intriguingly, we saw waves of reversed impulse propagation in Alzheimer’s disease brain, notably in mesiotemporal and periventricular structures, thus implying marked alterations in perivascular CSF dynamics in the Alzheimer’s disease brain. Note, that apparent reversed impulse propagation between regions may originate from non-trivial sources, such as latency shifts or slowing pulse in a region compared to neighbouring areas.

The functional MRI BOLD signal originates in T_2_*-weighted signal like MREG_BOLD_ signal both from intravascular (2/3) and perivascular (1/3) water protons.^24^ The water proton spins reflect shielding effects of oxy- and deoxyhaemoglobin in the vascular compartment, as well as blood volume and other physiological factors. The most common use of the BOLD signal is to depict slow haemodynamic changes coupled to changes in regional brain activity.^41^ In addition to the slow changes in haemodynamic BOLD signal level, accumulating evidence show fast signal variance arising in association with aortic pulse wave velocity and Alzheimer’s disease.^22^^,^^23^

Recent high-speed BOLD scanning techniques, like the MREG sequence used in this study,^28^ enable exact separation of faster physiological activity such as traversing cardiorespiratory impulses from conventional BOLD signal baseline changes, without signal aliasing.^16–18^^,^^28^ While researchers have tended to view the cardiorespiratory impulses as noise obscuring the BOLD signal, in more recent conceptions, cardiovascular impulses actually perform a key homeostatic function in pushing water along vascular and perivascular CSF spaces, thus moving metabolites and clearing waste material from the brain by the convective transport.^12–15^ The arrival of a cardiac pressure impulse transiently reduces the MREG signal due to the sudden perturbation of spin coherence among water protons both within arteries/arterioles but also in perivascular spaces. Only functional MRI techniques fast enough to fulfil the Nyquist theorem at minimum two images per heartbeat (i.e. repetition <300 ms in practice) can capture these short-lived arterial water spin perturbations.^17^

Optical flow analysis of MREG signal in the healthy brain indicates that the cardiovascular impulse becomes absorbed into a propagating pressure wave affecting water molecules that traverse along the arterial pathways in a narrow speed range of 15–35 mm/s through the brain.^16^ As known from *in vivo* mouse studies, the arterial impulse acts as a force driving the perivascular and intravascular water convection.^14^ However, in the brain of patients with Alzheimer’s disease, the impulse speed *v*_rms_ and the latency were higher in more peripheral vascular territories and were lower close to major branches of the cerebral arteries (Figs 1B and 3A). The propagation differences were observed to maximize along the axis of the large arteries and respected the vascular tree territories. In physical terms, increased impulse propagation speed reflects greater hydrostatic pressure and arterial wall tension, reduced pulsatility of the affected arterial wall with narrowed lumens and/or perivascular spaces in the peripheral small arterial/arteriolar trunks.^9–11^^,^^42^^,^^43^ Intriguingly, the increased blood impulse propagation speed has been found to be a predictive factor of Alzheimer’s disease development in systematic evaluations of haemodynamics.^11^

We found a considerable (47.6%) overlap between regions typically showing amyloid-β accumulation in early Alzheimer’s disease^40^ with zones of increased impulse propagation speed occurring at the very moment of the arterial impulse arrival. Accumulation of amyloid-β within the vascular wall structures of the blood–brain barrier and of tau filaments in the perivascular space induce 16–65% narrowing of microvessel diameter.^42–44^ This could indicate that the peripheral arterial structures with the greatest amyloid-β accumulation in the intramural and perivascular parts have the most restricted impulse propagation.^9^^,^^42–44^ Accordingly, the observed speed changes suggest that there may be increased arterial wall tension at the margins and reductions in the proximal cerebrovascular tree in patients with Alzheimer’s disease. Alternatively, since PET detects more parenchymal than intravascular amyloid-β, overlapping findings may reflect paravascular rather than intravascular amyloid-β involvement in the process.

In addition to relative changes in speed *v*_u_, we also report intriguing reversals of the direction of cardiovascular impulse propagation in mesiotemporal and periventricular brain regions of the Alzheimer’s disease group. Stiffened arteries and hypertension increase likelihood of reversed impulses, also known as reflective waves of blood pressure.^7^^,^^11^ Increased reflective indexes, especially in more peripheral carotid rather than proximal aorta, have been shown to be linked to cognitive decline, brain atrophy and amyloid-β depositions contributing to Alzheimer’s disease pathology.^7^^,^^11^^,^^44^ In this study, we saw propagation reversal between the zones of increased and reduced propagation speed, also supporting the notion that greater tension in vessel walls at the periphery of vascular territories results in narrowing of the perivascular space by vascular wall pathology.

Beginning with the description of vascular sclerosis in the case report by Alois Alzheimer in 1907,^45^ blood vessel pathology has been strongly linked to Alzheimer’s disease pathology. The vascular protein aggregates also reduce vessel pulsatility, thus impeding also perivascular flow, which could be a mechanism further explaining the altered propagation speeds detected in this study.^9^^,^^11^^,^^42^ A cessation/reversal of normal perivascular CSF convection could be permissive to increased accumulation of toxic metabolites and protein aggregates, and vessel wall inflammation in Alzheimer’s disease.^7^^,^^14^^,^^15^^,^^43^^,^^44^ Reversed pulse propagation and increased pulsation speeds could impose localized hydrostatic stress on blood vessel walls, possibly accounting for the recently reported blood–brain barrier damage in Alzheimer’s disease.^46^^,^^47^ The zones with reversed impulse propagation were mostly overlapping with the hippocampi, where the increased blood–brain barrier permeability evoked PDGFβ-R (platelet-derived growth factor receptor) leakage in connection to (peri)vascular amyloid deposits, a finding recently detected both in animal models and human Alzheimer’s disease studies.^15^^,^^46–49^ Thus, we propose that the reversed pulse wave propagation in mesiotemporal and periventricular areas of Alzheimer’s disease patients are likely to cause shearing strain on the perivascular conduits in the externa limitans of the blood–brain barrier.^47^

Despite its new technological advances, this study has limitations. While translational and rotational head motion detection using standard motion correction tool FSL MCFLIRT^31^ showed no significant differences between the groups, localized non-linear motion artifacts stemming from cardiorespiratory pulsations, especially in the subcortical regions, is a confounding factor in the results. Furthermore, the relatively large MREG voxels also introduce partial volume effects which can also confound the results. Significant grey matter atrophy is present in this Alzheimer’s disease population, partly overlapping with altered cardiovascular pulse propagation speed in temporoparietal areas. While spatial correlations between the group level grey matter atrophy map and our results were low, individual level grey matter atrophy may also be another confounding factor in the results as we were not able to use the individual grey matter atrophy maps as regressors. Future work is needed to improve spatial resolution for capturing non-linear cardiorespiratory motion and minimizing partial volume effects.

In conclusion, ultrafast BOLD signal data analysis indicates marked perturbation of cardiovascular brain impulse propagation along the cerebral arterial territories of Alzheimer’s disease patients. Findings vary along the position in the vascular tree, such that proximal arteries seem to be dilated, whereas peripheral vascular territories showed increased impulse speed possibly linked to narrowed peri/intravascular patency. We also detect directional reversals of the impulse propagation in intermediate zones of the vascular trees proximal to the mesiotemporal and periventricular areas, which is consistent with partial occlusions in the peri/intravascular spaces towards the periphery of the arterial trees; these areas seem to match regions typically showing elevated amyloid-β accumulation and blood–brain barrier pathology in Alzheimer’s disease. The abnormal impulse propagation may thus underly impaired vascular flow, which is in turn relevant to impairment of the perivascular CSF pathway for clearance of waste and metabolites that predisposes to neurodegeneration and cognitive decline in Alzheimer’s disease.

## Supplementary Material

awab144_Supplementary_Data

## Abbreviations

ACA: anterior cerebral artery
BOLD: blood oxygenation level-dependent
MREG: magnetic resonance encephalography
