# Erratum to: Cardiovascular brain impulses in Alzheimer’s disease

**DOI:** 10.1093/brain/awab298

**Published:** 2021-09-22

Zalán Rajna, Heli Mattila, Niko Huotari, Timo Tuovinen, Johanna Krüger, Sebastian C. Holst, Vesa Korhonen, Anne M. Remes, Tapio Seppänen, Jürgen Hennig, Maiken Nedergaard, Vesa Kiviniemi. Cardiovascular brain impulses in Alzheimer’s disease. *Brain*. 2021;144(7):2214–2226. doi:10.1093/brain/awab144

The publisher apologizes for not including the video abstract and for an error in uploading the Supplementary material. These have been corrected.

