# Supplementary material for: Cardiovascular brain impulses in Alzheimer’s disease
Source: Brain. 2021 Mar 31;144(7):2214–26. doi: 10.1093/brain/awab144 (PMC8422353; doi:10.1093/brain/awab144)
Supplement: awab144_Supplementary_Data [file awab144_Supplementary_Data.zip › awab144_Supplementary_Data/supplementary_material.pdf]

Cardiovascular brain impulses in Alzheimer's disease

Supplementary material

Zalán Rajna*, Heli Mattila, Niko Huotari, Timo Tuovinen, Johanna Krüger, Sebastian C. Holst, Vesa Korhonen, Anne M. Remes, Tapio Seppänen, Jürgen Hennig, Maiken Nedergaard, and Vesa Kiviniemi*

*Corresponding authors: `zalan.rajna@oulu.fi`, `vesa.kiviniemi@oulu.fi`

Contents

Supplementary Figures and Tables

- **Supplementary Fig. 1**: Example frame of MREG data.
- **Supplementary Fig. 2**: Illustration of optical flow analysis.
- **Supplementary Fig. 3**: Voxelwise directions of maximal difference in Alzheimer's disease at $t = 0.0$ s.
- **Supplementary Fig. 4**: Full brain coverage of v_{rms} changes in Alzheimer's disease as in Fig. 3A.
- **Supplementary Fig. 5**: Full cardiac cycle of faster and slower pulse propagation speed (v_u) in Alzheimer's disease for planes intersecting at MNI $[0, -25, -11]$ as in Fig. 5.
- **Supplementary Fig. 6**: Full cardiac cycle of reversed pulse propagation direction in Alzheimer's disease for planes intersecting at MNI $[30, -25, -11]$ as in Fig. 6A.
- **Supplementary Table 1**: Subjects included in this study.
- **Supplementary Table 2**: Spatial correlations with gray matter atrophy map.

Supplementary Videos and Data (not included in this document)

- **Supplementary Video 1 (`video1.mp4`)**: MREG data of 0.9 s cardiac cycles (control, AD); speed magnitudes (v_{rms}), and the difference in v_{rms} with significant parts highlighted as in Fig. 3A.
- **Supplementary Video 2 (`video2.mp4`)**: Directions of maximal difference in Alzheimer's disease (AD); areas of faster, slower, and reversed pulse propagation as in Figs 4-6A.
- **Supplementary Data 1 (`AD_faster.nii.gz`)**: Difference in propagation speed (v_u in mm/s) of areas with faster pulse propagation in Alzheimer's disease.
- **Supplementary Data 2 (`AD_slower.nii.gz`)**: Difference in propagation speed (v_u in mm/s) of areas with slower pulse propagation in Alzheimer's disease.
- **Supplementary Data 3 (`AD_reversed.nii.gz`)**: Difference in propagation speed (v_u in mm/s) of areas with reversed pulse propagation in Alzheimer's disease.
- **Supplementary Data 4 (`AD_directions.nii.gz`)**: Voxelwise directions of maximal difference in Alzheimer's disease at $t = 0.0$ s calculated from all $17,393$ cardiac cycles.

Supplementary Figures

An example of MREG time frame after reconstruction and masking but before further processing is presented in Supplementary Fig. 1.
Schematic explanation of wavefront propagation calculated as optical flow in 3D is shown in Supplementary Fig. 2.
In Supplementary Fig. 3 we show that at the reference time point of cardiac impulse arrival ($t = 0.0$ s), cardiac cycle length hardly alters directional maps presented in Fig. 4.
Full brain coverage of the speed magnitude (v_{rms}) differences of pulse propagation speed between Alzheimer's disease and control groups are presented in Supplementary Fig. 4.
The full cardiac cycles for Figs 5 & 6A are shown in Supplementary Figs 5 & 6, respectively.

Supplementary Tables

Supplementary Table 1: **Subjects included in this study.** Sex, age, MMSE score, Alzheimer's disease (AD) duration, and rigid displacement (absolute motion) from reference frame of subjects included in this study.

Subject group	Number (females)	Age (years)	MMSE score	AD duration (years)	Motion (voxel)
Control	26 (11)	57.4±5.7	28.6±1.3	n.a.	0.18±0.14
AD	31 (18)	60.5±4.8	22.3±6.3	3.5±2.2	0.24±0.14

Supplementary Table 2: **Spatial correlations with group level gray matter atrophy map.** Spatial correlation coefficients (`fslcc`) between the Alzheimer's disease (AD) gray matter atrophy map (voxel-wise group level P-value map as "$1 - P$") and the following results in separate rows: voxel-wise v_u speed differences (mm/s) between groups (AD vs. controls) in areas of faster, slower and reversed Alzheimer's disease pulse propagation; and v_u voxel-wise group difference statistic (p-value map as "$1 - P$") regardless of difference type, i.e. the combination of the three areas above. Each column represents a cardiac phase (timing) compared to pulse arrival into the brain.

Result map	-0.5 s	-0.4 s	-0.3 s	-0.2 s	-0.1 s	0.0 s	0.1 s	0.2 s	0.3 s
AD faster (mm/s)	0.0067	0.0089	0.0012	0.0117	0.0180	0.0721	0.0192	0.0085	0.0055
AD slower (mm/s)	0.0043	0.0049	0.0058	0.0002	0.0455	0.0496	0.0732	0.0116	0.0156
AD reversed (mm/s)	0.0081	0.0045	0.0297	0.0311	0.0384	0.0717	0.0219	0.0163	0.0036
v_u *difference statistic (1-P) three areas above combined*	0.0136	0.0194	0.0685	0.0720	0.0723	0.1637	0.1318	0.0168	0.0271

Supplementary Videos

Technical information: 3840×2160 (4K) resolution, H.264/AVC codec, 4 Mb/s, 30 fps.

- **Supplementary Video 1 (`video1.mp4`)**: MREG data of 0.9 s cardiac cycles (control, AD); speed magnitudes (v_{rms}), and the difference in v_{rms} with significant parts highlighted as in Fig. 3A.
- **Supplementary Video 2 (`video2.mp4`)**: Directions of maximal difference in Alzheimer's disease (AD); areas of faster, slower and reversed pulse propagation as in Figs 4-6A.

Supplementary Data

Difference in propagation speed (v_u in mm/s) of areas with faster, slower, and reversed pulse propagation in Alzheimer's disease, are provided in NIfTI format as `AD_slower.nii.gz`, `AD_faster.nii.gz`, and `AD_reversed.nii.gz`, respectively.
We also provide NIfTI data (`AD_directions.nii.gz`) of Fig. 3B: the voxelwise directions of maximal difference in Alzheimer's disease at $t = 0.0$ s calculated from all $17,393$ cardiac cycles.

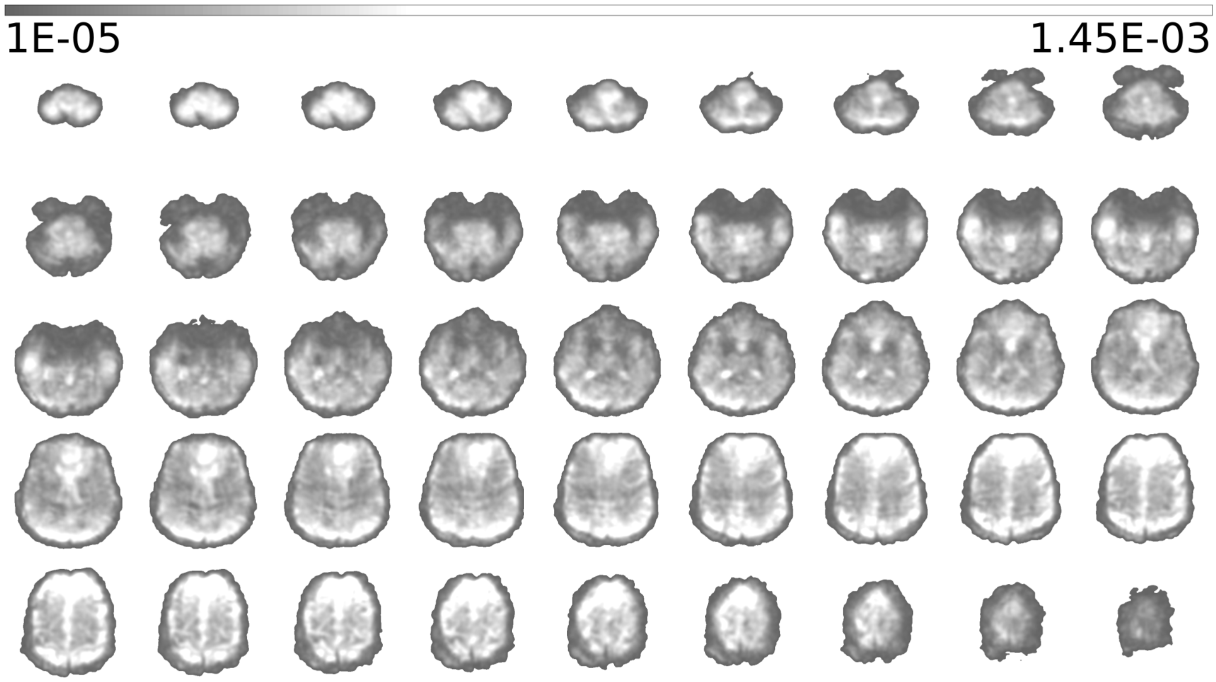

Supplementary Figure 1: **Example frame of MREG data.** The frame was taken from a healthy subject and presents an MREG time frame after reconstruction and masking, but before any further processing.

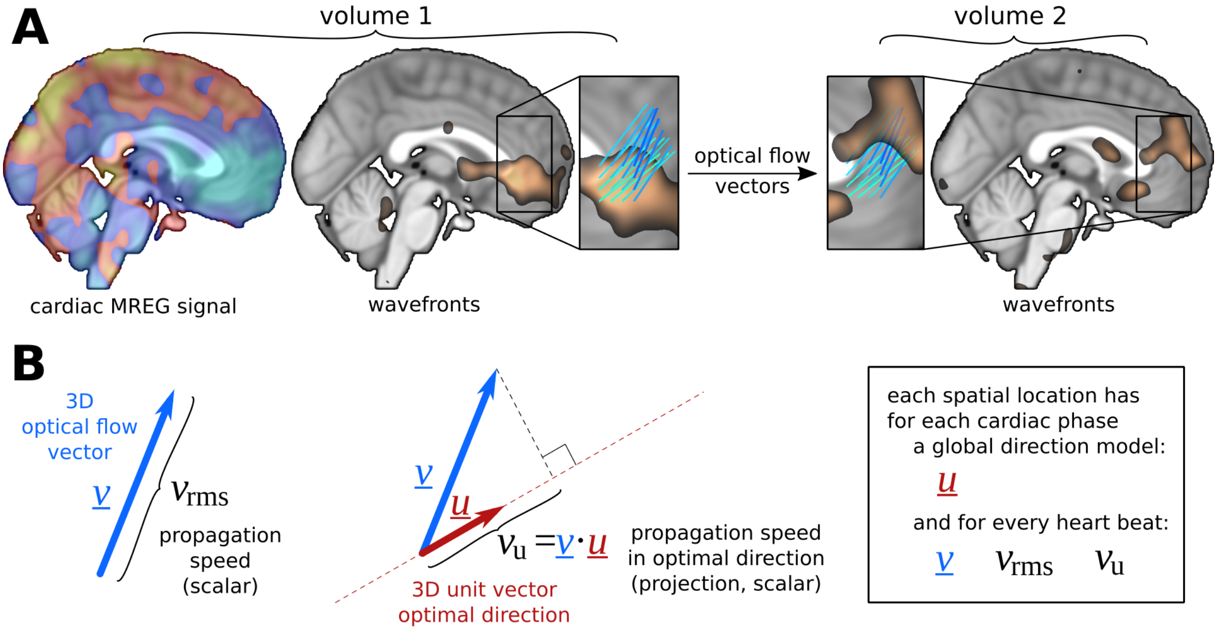

Supplementary Figure 2: **Illustration of optical flow analysis.** (**A**) Cardiac MREG signal, and the extracted wavefronts (local minima) from volume 1. Their propagation calculated as optical flow vectors, and the wavefronts of volume 2. In this example the propagation direction is the superposition of a coronal (blue) and an axial (green) component, and the color of the vectors is weighted between those accordingly. (**B**) Illustration of an optical flow vector $\underline{\mathbf{v}}$, the vector length (magnitude, L2 norm) v_{rms} (c.f. results in Fig. 3), unit vector $\underline{\mathbf{u}}$ into which direction the group differences are maximal (c.f. results in Figs 4 and 5A), and v_u as the projection of $\underline{\mathbf{v}}$ on $\underline{\mathbf{u}}$ which means the length of $\underline{\mathbf{v}}$ in the direction of $\underline{\mathbf{u}}$ (c.f. results in Figs 5B and 6A). Direction $\underline{\mathbf{u}}$ is a result of inter- and intra-subject optimization (c.f. Equation 1) and is also temporally global, i.e. it is unique for each cardiac phase and spatial location in the whole dataset. In contrast, vector $\underline{\mathbf{v}}$, together with v_{rms} and v_u derived from $\underline{\mathbf{v}}$ are in addition unique for each heartbeat and time point as well.

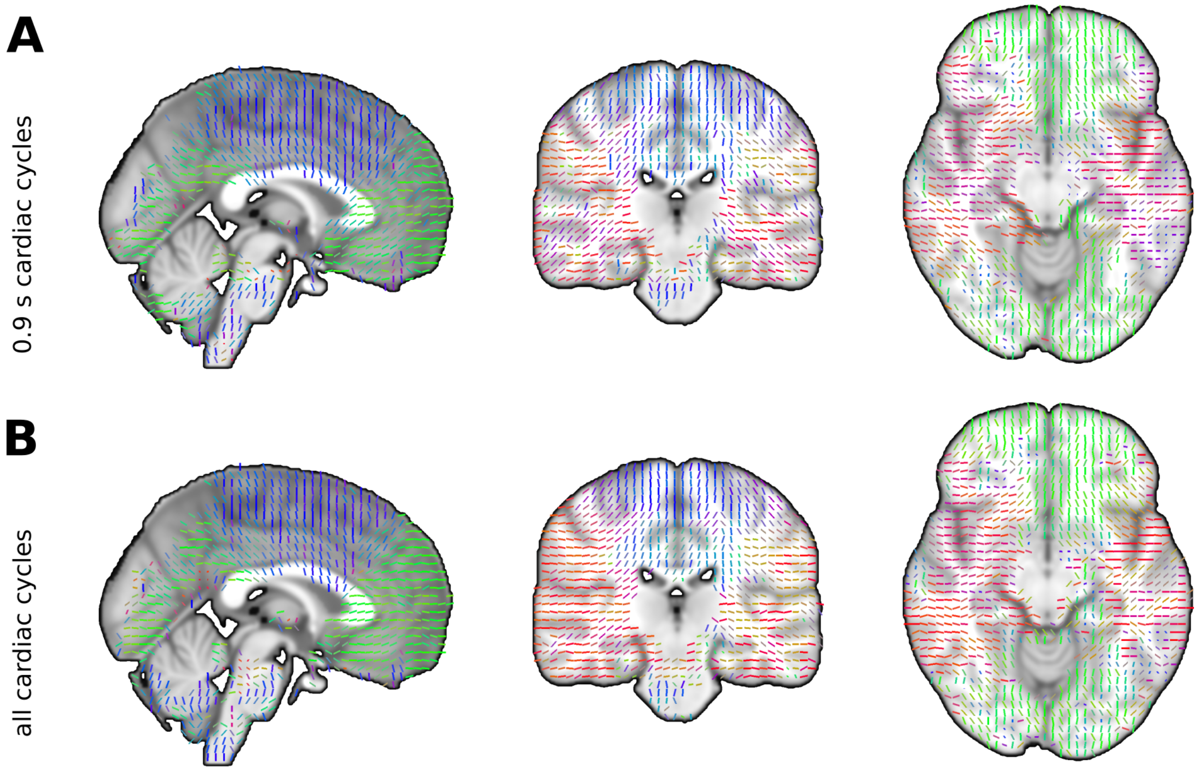

Supplementary Figure 3: **Voxelwise directions of maximal difference in Alzheimer's disease at $t = 0.0$ s.** Selected 3D planes (MNI: $[0, -25, -11]$) showing (**A**) the 0.9 s long cardiac cycles, same as in Figs 5 & 6A, and Supplementary Figs 5A & 6A, and (**B**) all $17,393$ cardiac cycles recorded.

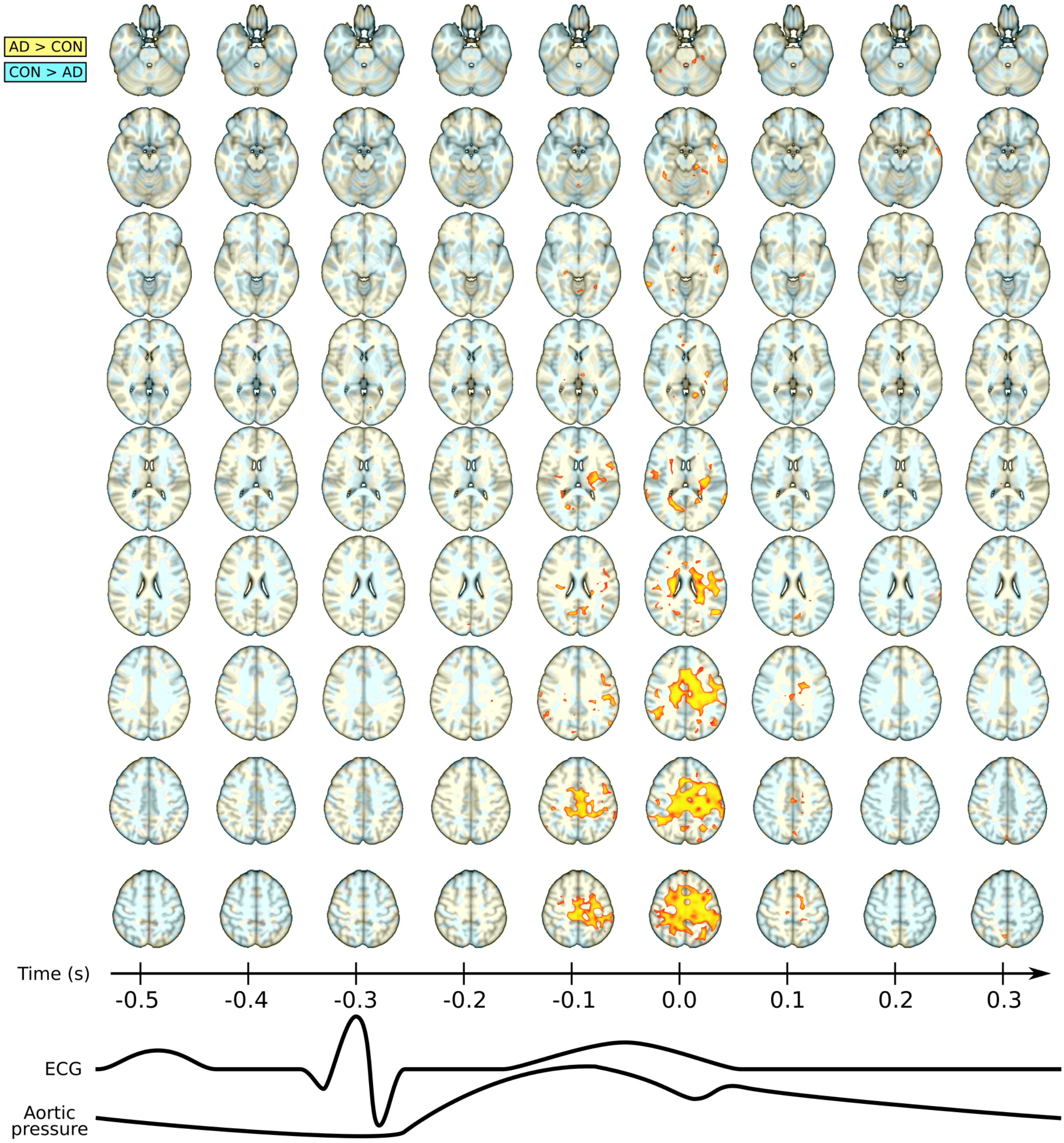

Supplementary Figure 4: **Full brain coverage of $v_{\mathbf{rms}}$ changes in Alzheimer's disease as in Fig. 3A.**

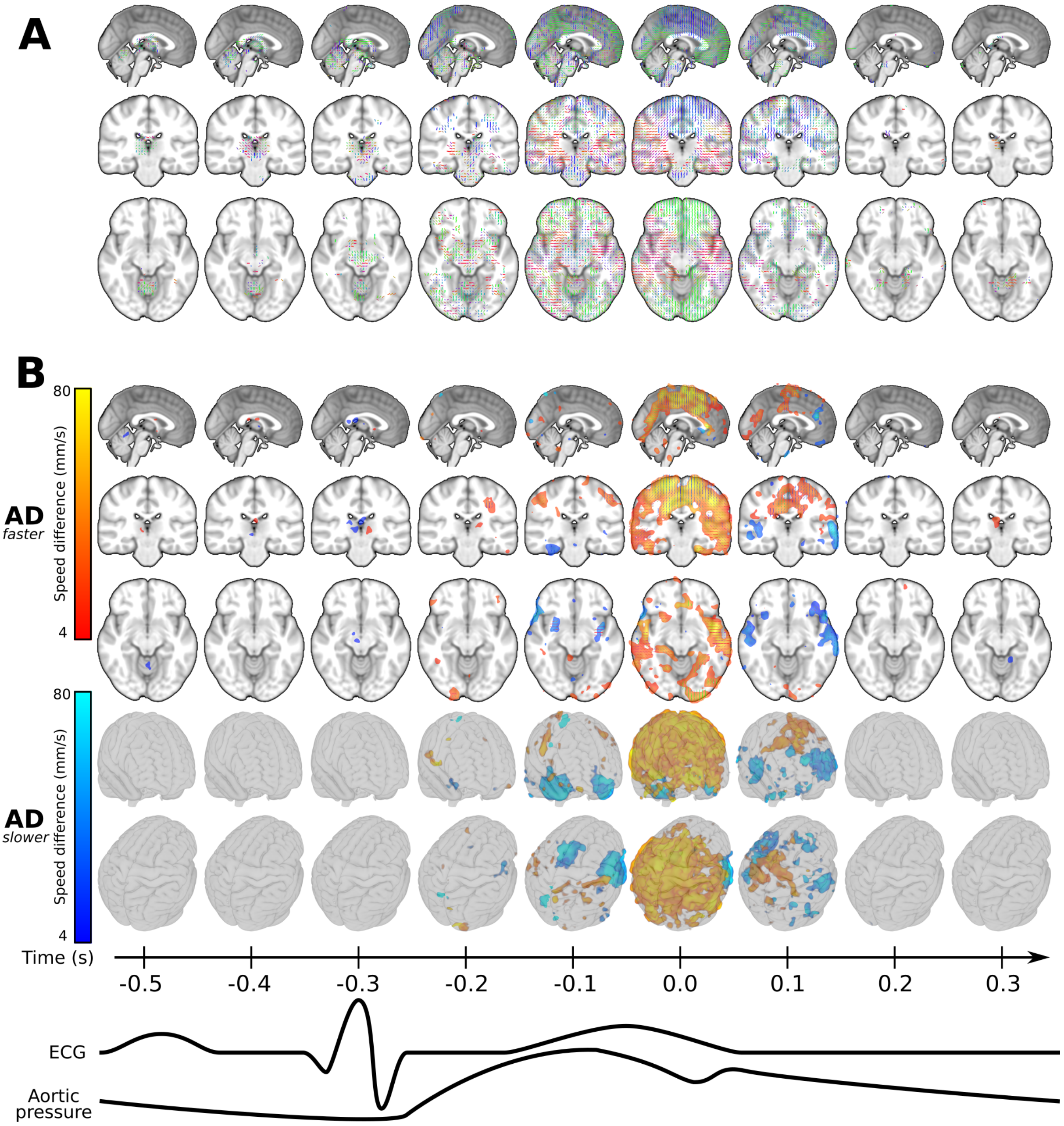

Supplementary Figure 5: **Full cardiac cycle of faster and slower pulse propagation speed** (v_u) **in Alzheimer's disease at MNI** $[0, -25, -11]$ **as in Fig. 5.** (**A**) The directions and (**B**) the covered areas.

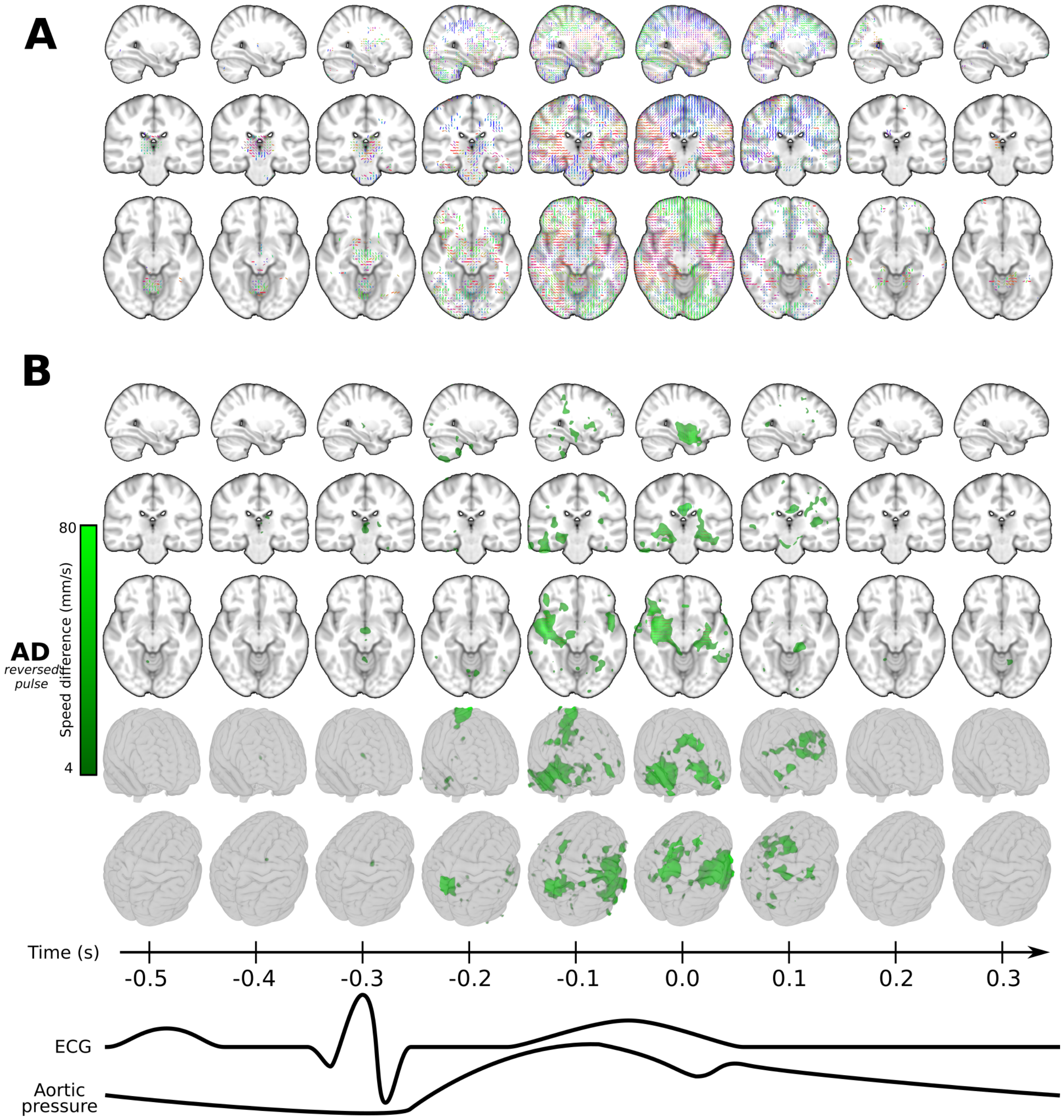

Supplementary Figure 6: **Full cardiac cycle of reversed pulse propagation direction in Alzheimer's disease at MNI** $[30, -25, -11]$ **as in Fig. 6A.** (**A**) The directions and (**B**) the covered areas.
